# HPV Vaccination Among Young Adult Women: A Perspective From Appalachian Kentucky

**DOI:** 10.5888/pcd10.120183

**Published:** 2013-02-07

**Authors:** Laurel A. Mills, Katharine J. Head, Robin C. Vanderpool

**Affiliations:** Author Affiliations: Katharine J. Head, University of Kentucky College of Communications and Information Studies, Lexington, Kentucky; Robin C. Vanderpool, University of Kentucky College of Public Health, Lexington, Kentucky.

## Abstract

**Introduction:**

Few studies have assessed barriers to human papillomavirus (HPV) vaccination uptake and adherence, particularly among women of Appalachian Kentucky, a population with higher rates of cervical cancer, lower rates of HPV vaccination, and lower socioeconomic status compared with the rest of the nation. The objective of this study was to address women’s reasons for declining the HPV vaccine and, among women who initiated the vaccine series, barriers to completion of the 3-dose regimen.

**Methods:**

We recruited 17 women aged 18 to 26 from a Federally Qualified Health Center who participated in in-depth, semistructured telephone interviews. All interviews were audio-recorded and transcribed verbatim; analysis of the interview transcripts was an iterative process conducted by all 3 authors.

**Results:**

We identified 3 primary barriers: 1) a knowledge gap wherein women are both uninformed and misinformed about cervical cancer, HPV, and the HPV vaccine, all of which affect vaccination behaviors; 2) environmental and tangible barriers (transportation and prioritizing health over other responsibilities such as child care, work, and school); and 3) ambiguous information sources, which contribute to misinformation and subsequently affect vaccination decisions.

**Conclusion:**

Health professionals should use clear and purposeful communication about how cervical cancer develops, the purpose and safety of the HPV vaccine, and the necessity of completing the 3-dose series. Health promotion campaigns and services tailored for young women in Appalachian Kentucky that focus on increasing knowledge and eliminating barriers are needed.

## Introduction

Prevention of human papillomavirus (HPV) infection and subsequent development of cervical cancer is achievable through vaccination against HPV types 16 and 18. Two vaccines are available, both given as 3 doses over 6 months and approved for women aged 9 to 26; however, vaccination rates remain low for adolescent girls and young adult women (1,2).

Research on HPV vaccination has focused on perceived barriers to vaccine initiation for parents of adolescent girls (3,4). Few studies have assessed barriers to HPV vaccination uptake and adherence, particularly among women of Appalachian Kentucky, a population with higher rates of cervical cancer, lower rates of HPV vaccination, and lower socioeconomic status compared with the rest of the nation (Table) (2,5–10). Scant research has addressed young adult women who were not vaccinated as adolescents, may be uninsured or underinsured, are ineligible for the federal Vaccines for Children Program, or are not the primary targets of mainstream HPV vaccine marketing campaigns (11,12). 

**Table:** Cervical Cancer–Related Disparities, Appalachian Kentucky Compared With the United States

Item Compared	Area, %			

	United States	Kentucky	Appalachian Kentucky	Kentucky River Area Development District^a^
Cervical cancer incidence rate^b^	8.0	8.9	9.8	10.4
Cervical cancer mortality^b^	2.4	2.9	3.5	Unstable
No cervical cancer screening (Pap test) in past 3 years^c^	18.3	17.2	22.2	25.7

Abbreviation: HPV, human papillomavirus; Pap, Papanicolaou.

a The Kentucky River Area Development District includes Breathitt, Knott, Lee, Leslie, Letcher, Owsley, Perry, and Wolfe Counties.

b Age-adjusted, per 100,000 women (2005–2009).

c For women aged 18 or older.

Young women in Appalachian Kentucky misunderstand HPV and the HPV vaccine, which may undermine their vaccination uptake (14). Women living in Appalachian Kentucky may also face environmental constraints such as cost, limited transportation, geographic distance, and lack of insurance (15,16). Sources of social influence that make up one’s perceived norms, such as religion and family, can moderate women’s intentions to be vaccinated; familial influences may play a role in adult women’s vaccination intentions (14,17,18). 

This qualitative study builds on previous quantitative findings, which indicate that women in Appalachian Kentucky are significantly less likely to return for subsequent doses of the HPV vaccine than urban Kentucky women (5). The objective of this study was to addresses women’s reasons for declining the HPV vaccine and, among those who initiated the vaccine series, barriers to completion of the 3-dose regimen.

## Methods

The study was guided by the Integrated Behavioral Model (IBM), which is an approach to understanding theoretical constructs relevant to knowledge, attitudes, perceptions, behavioral intention, and behavior (13); several IBM constructs may play a role in HPV vaccination. The IBM posits that knowledge is required for behavior performance and that environmental constraints serve as barriers, impeding behavior. Given the IBM’s focus on these constructs and its previous application in health behavior research (19,20), we used it as a framework for this study. We used in-depth, semistructured telephone interviews. The University of Kentucky institutional review board approved study procedures.

### Recruitment and participants

On the basis of consultation with the medical director of a regional Federally Qualified Health Center (FQHC) in Appalachian Kentucky, investigators designed a study protocol that identified female clinic patients aged 18 to 26, through a medical chart review, who had either declined the HPV vaccine or had started the vaccine series but failed to complete doses 2, 3, or both in the required time. We asked to review medical charts from March 2008 through September 2009 because an HPV vaccine promotion, which provided the vaccine free to eligible women, occurred during this time. FQHCs provide care for medically underserved people for free or on a sliding scale; more than 275,000 Kentuckians, 63% of them women, used an FQHC in 2010 (21). Approximately 600 women in the targeted age group are served annually at this FQHC.

All eligible women (N = 150; 65 declined the vaccine, and 85 received doses 1 or 2 only) were invited to participate in the telephone interviews. Despite multiple recruitment attempts (mail and telephone), only 18 women enrolled; recruitment was challenging because of the study population’s transient nature (ie, high rates of disconnected telephone numbers and invalid mailing addresses). One interview was excluded from analysis because of inaccurate medical records; the final sample was 17 women. All of the women were white, and the mean age was 22 years; only 2 women had health insurance. Eight study participants were married. Nine of the women initiated the vaccine series, and 8 declined the vaccine.

### Procedures

Telephone interviews that lasted approximately 30 minutes were conducted between June through August 2010; each participant was compensated $30. Participants provided informed consent before starting and recording the interviews. Participants answered every interview question (including responses of “I don’t know”). The interview guide (Appendix) was informed by previous literature (3,22,23), the quantitative findings of Crosby et al (5), the IBM framework (knowledge, barriers, and social influence), and consultation with the clinic medical director.

The interviewer (L.A.M.) read the transcripts to verify accuracy and remove any personal identifiers. Pseudonyms were created for participants to designate vaccination status; participants who declined the vaccine were given a final initial identifier of “D” (eg, Rebecca D), and participants who received at least 1 dose were given a final initial identifier of “V” (eg, Whitney V).

### Data analysis

All 3 authors analyzed the interview transcripts via an iterative process. Using the IBM framework and literature on cervical cancer, HPV, and HPV vaccination, we developed a codebook that was used to initially code the interviews. All 3 authors read each transcript line by line using the initial coding frame. The authors then met to discuss the initial coding and discussed divergent or unique findings, which led to the generation of a final codebook (still informed by the IBM framework). The first author (L.A.M.) recoded the transcripts using the final codebook, gathering key examples for each emerging finding.

## Results

Both groups of women, those who declined the vaccine and those who initiated the vaccine, had similar knowledge levels related to cervical cancer, HPV, and HPV vaccination and similar environmental and social normative barriers. Participants discussed 3 barriers to vaccination, which were consistent with the IBM’s behavioral antecedents: 1) knowledge barriers to HPV vaccination, with a distinct difference between misinformation and being uninformed; 2) environmental and tangible barriers to HPV vaccination such as transportation and everyday responsibilities; and 3) social and normative influences on HPV vaccination.

### Knowledge barriers to HPV vaccination: misinformed versus uninformed

Participants were misinformed as well as uninformed about cervical cancer, HPV, and the HPV vaccine. *Uninformed* is defined as lacking knowledge whereas *misinformed* is defined as having incorrect knowledge. Four women were uninformed about cervical cancer; they answered, “I don’t know” when asked about cervical cancer and how it develops. Women also lacked knowledge about HPV; 5 women responded “I don’t know” to HPV questions, 4 women were uninformed about HPV transmission, and 2 women who were unvaccinated had never heard of the HPV vaccine. Some women were misinformed about these issues. For example, when asked about the causes of cervical cancer, Susan D stated it develops “from being really [sexually] active” and “tissue scarring.” Rebecca D said she has “always heard tanning beds” can cause cervical cancer. Brenda D said, “I’ve actually heard that using [a] douche can cause cervical cancer.” Whitney V said cervical cancer “could be hereditary, possibly.” These examples represent the range of misinformation about cervical cancer, espoused by both vaccinated and unvaccinated women.

Women in this sample were misinformed about HPV, even confusing it with cervical cancer and the HPV vaccine. When asked about HPV, Rebecca D said it is “a shot that prevents cervical cancer”; Whitney V said HPV is “a type of cancer.” Susan D shared that HPV “is a disease that can kill you.” Finally, some women were unsure about the vaccine dosing schedule; for example, Leah V said, “When I took the first dose, I didn’t know there was 2 more doses.”

### Environmental barriers

Environmental barriers to medical services are not uncommon in this region. Three women indicated that transportation was problematic. Elizabeth V shared that “me and my fiancé, neither one have a driver’s license, so we don’t have a car. . . . We’re dependent on my mother to take us [places].” Monica V stated, “I don’t drive; my husband’s the only one that drives, and he works full time.” Felicia V said she doesn’t “have any transportation,” and this was a barrier for getting subsequent HPV doses. Even women who had transportation indicated that lack of transportation could be problematic for their peers to return for follow-up vaccinations.

Many young women also shared that their everyday responsibilities prohibited them from completing the HPV vaccine series. A major barrier was child care. Elizabeth V shared that she was unable to return for subsequent doses because “My oldest son is going to school, and me, I had to take care of a newborn.” Likewise, Jane V said, “I want to get this [HPV vaccination] done, but I always put my daughter first. So if she’s sick or has to go to the doctor, I always take her before I do myself.” School and work responsibilities were also barriers to series completion for these women. Leah V explained, “I work 6 days a week . . . from 9 a.m. until 8 p.m. in the evening, so it’s hard” to make time for HPV vaccination. School, work, and child care responsibilities are common in this population of young women, making this age group difficult to reach.

Despite challenges to vaccination, women were supportive of making the vaccine more widely available in the community at places like the local Walmart, community college, and community festivals. For example, when asked if providing the HPV vaccine at different community locations could help other women like her to receive the vaccine and return for subsequent doses, Elizabeth V said “that would catch a lot of women in this age group.” Additionally, Jane V indicated, “I would definitely do something like that. . . . I think that’s an excellent idea.” Shannon D also agreed, saying “that would be good” to deliver the vaccine in the community. Diane D suggested that “someone [should be] there to counsel on the risks and benefits” as well. Overall, environmental barriers related to everyday responsibilities and transportation issues were common; however, there was support for opportunities to give the vaccine in the community.

### Sources of social influence

Religious beliefs did not influence vaccination decisions in this sample. Furthermore, 12 women, regardless of vaccination status, did not discuss their vaccination decision with family. When women did discuss their vaccination decisions with family, they were mostly neutral or positive. Whitney V remembered, “I spoke with my family about it and my husband.” Similarly, Amanda V discussed her decision to get vaccinated with her family and they “agreed that it would be a good precaution.” There was 1 instance of a family member bringing up the vaccine to a young woman. Sarah D shared, “My mom actually had mentioned [the HPV vaccine] to me before . . . when she found out she had cervical cancer . . . but I never [personally] asked [about it].” In this case, the positive conversation did not lead to vaccination.

Although known social influences such as family and religion did not affect these women’s decision to vaccinate, ambiguous sources from which these women received information about cervical cancer, HPV, and the HPV vaccine were commonly mentioned. Many women referenced “they” when discussing their information sources. “They” make up a piece of Appalachian Kentucky women’s social and normative network — a mix of cultural perceptions and health information obtained from family, friends, mass media, and health care providers. For instance, Mary V shared that she was instructed to stop taking the HPV vaccine because “there’s a big rumor . . . going around about them saying [the shots] done something; you couldn’t have kids.”

Brenda D, who believed that douching can cause cervical cancer, went on to say that “they told me not to do that [get the HPV vaccine] because it could cause [cervical cancer], they said so.” Another instance of ambiguous information sources was evidenced by Ashley V, who said she heard the HPV vaccine “ caused a lot of people to die in Arizona. . . . A lot of people were taking the shot got sick and died,” and she heard this from “other women.” When asked what local health clinics should do to increase vaccine uptake and adherence for young women like her, Ashley V believed it was the health care providers’ responsibility to inform young women about these health issues and clear up any misconceptions. She said that providers should “make sure that they’ve answered all their questions . . . [that] they have laid out the facts.” 

## Discussion

As supported by the IBM, we found 3 major barriers to HPV vaccination. First, we found a knowledge gap among both unvaccinated and partially vaccinated women related to cervical cancer, HPV, and the HPV vaccine, which affected women’s decisions to vaccinate or continue vaccinating, confirming previous research (23,24). Although several of our study participants were simply uninformed about cervical cancer, HPV, and the vaccine, some women were misinformed.

Second, tangible barriers prevented these women from receiving the vaccine or completing the series. Women identified lack of transportation as a barrier. Busy schedules and other priorities such as family, work, and school were also barriers. The young women in our study are starting their adult lives, going to school, getting married, and having children, often while living on a constrained budget and in an area of the country where they may be geographically isolated from a health clinic. These factors combine to create a hard-to-reach population that is less likely to return to clinics for subsequent vaccine doses. To increase vaccine uptake and adherence, public health practitioners in this area should design programs that partner with communities to deliver this vaccine. Community-based approaches for reducing cancer disparities in Appalachian Kentucky show that local residents can provide insight into local health issues and possible locally tailored solutions (25). Others have found success in bringing services to communities with mobile mammography (26) and offering influenza vaccinations at alternative locations (27).

Third, we found social and normative influences as barriers. Some findings were unexpected: the women in our sample denied that religion and family influenced their vaccination decision, despite research indicating the opposite (3). These sources of influence may fade or may not be as salient for women making health decisions as adults (28). The ambiguous sources from which this sample of Appalachian Kentucky women received information about cervical cancer, HPV, and the HPV vaccine present challenges for providers. Obtaining information from people in one’s social network who are not medically educated and who are living in small, insulated communities may make these women susceptible to rumors about vaccination and other health issues (14). Providers must be vigilant in informing as well as correcting misinformation about cervical cancer, HPV, and HPV vaccination to encourage uptake and adherence in this population.

Limitations of this study include the small sample size; women in this age group are often transient, making recruitment challenging. Rural Appalachian women may be reticent to participate in research, which also may have affected our sample size (14,29). Finally, our results are applicable only to 1 region in Appalachian Kentucky.

Our results have implications for public health practitioners and health care providers, particularly in rural communities. We make 2 suggestions for future research and practice for health professionals working in Appalachian Kentucky and similar medically underserved communities. First, it is imperative that providers use clear and purposeful communication about how cervical cancer develops, the purpose and safety of the HPV vaccine, and the necessity of completing the 3-dose series. Second, the dual knowledge gap (being misinformed or uninformed or both) highlights the importance of doing formative research, especially for health educators, clinicians, and lay health advisors. Practitioners who are designing health promotion materials and programs must assess both gaps.

## Appendix. Interview Guides for Study on Human Papilloma Virus (HPV) Vaccines in Appalachian Kentucky

**A.** **Interview Guide for Women Who Received the First Human Papilloma Virus (HPV) Dose and Overdue for Doses 2 and/or 3**

**Knowledge of HPV and Cervical Cancer**

• What do you think causes cervical cancer?

• Have you heard of the human papillomavirus, or HPV? What do you know about HPV?

• Do you feel at risk for HPV infection or cervical cancer? How is HPV transmitted?

• Have you heard of the HPV vaccine, Gardasil? What do you know about it?

• Commercials — do they resonate in young women 18–26 in your area?

• Rumors, myths about the shot? Pain?

**Barriers and Perceptions**

• How many doses of the HPV vaccine have you received to date? (Probe: Are you overdue for dose 2 or dose 3?)

• Why did you agree to receive the first shot of the HPV vaccine? Please explain.

• Do you feel you needed more information on cervical cancer and HPV infection before you decide to receive the second and third doses? Please explain.

• What are the responsibilities or activities in your daily life that you feel prevented you from being able to get the second and third shots of the Gardasil vaccine? Please explain. Probe: If “forgetfulness” is mentioned, would reminders from the clinic have been helpful, such as in the form of a phone call or postcard in the mail? Probe: Is transportation an issue? Please explain.

• What would help you to remember to come back for the shots?

• Did you discuss with your family (mother, father, husband, boyfriend, grandmother) as to whether or not you should continue to receive the vaccine doses? How much did your family’s opinion impact your decision? Please explain.

• Did you feel that continuing the vaccine shots would have put you in conflict with your religious beliefs? Please explain.

• Would you have felt more comfortable receiving the second and third shots if the HPV vaccine had been around longer? (Relates to efficacy, safety, side effects). Please explain.

• Was it hard to remember that you needed to come back for booster doses? If so, please explain.

• How motivated were you to return for the booster doses and what detracted from your motivation levels? Please explain.

• Please describe the practical barriers that may have kept you from coming back to get the booster doses. Please explain.

• How could the health clinic help women get all 3 doses of the HPV vaccine? Please explain.

• How do you feel about taking the vaccine out into the community?

• How do you feel about the HPV vaccine being mandated for young girls for school?

**B.** **Interview Guide for Women Who Declined the HPV Vaccine**

**Knowledge of HPV and Cervical Cancer**

• What do you think causes cervical cancer?

• Have you heard of the human papillomavirus, or HPV? What do you know about HPV?

• Do you feel at risk for HPV infection or cervical cancer? How do you believe HPV is transmitted?

• Have you heard of the HPV vaccine, Gardasil? What do you know about it?

• Commercials — does it resonate with young women 18–26 there?

• Have you heard any rumors or myths about the shot? Pain?

**Barriers, Perceptions, and Cultural Issues**

• Do you want to get the HPV vaccine? Please explain.

• If yes, what would it [information, situation, influencer, etc] take for you to get the vaccine? Please explain.

• If no, describe what [information, situation, influencer, etc] could potentially change your mind about getting the vaccine. Please explain.

• Do you feel you have enough information to make a decision about whether or not to be vaccinated with the HPV vaccine? If not, what additional information could help you? Please explain.

• In what ways does your doctor influence you to be or not to be vaccinated against HPV? Please explain.

• In what ways would your mother, father, other relatives influence you to be or not to be vaccinated against HPV? Please explain.

• In what ways would your best friend influence you to be or not to be vaccinated against HPV? Please explain.

• Does cost affect your decision about being vaccinated against HPV? Please explain.

• Do your family values influence your decision to be vaccinated against HPV? Please explain.

• Do your religious beliefs affect your decision vaccinated against HPV? Please explain.

• Are the return trips to the clinic for the two booster doses of the HPV vaccine a barrier to being vaccinated against HPV? Please explain.

• What type of reminders do you think would work to help people to remember to get all 3 shots?

• What do you think about bringing the vaccine out into the community?

• How do you feel about mandating the HPV vaccine for schools in younger girls?

• How could the health care system help eliminate barriers to HPV vaccination among young women? Please explain.
